# How to improve regulatory practices for refurbished medical devices

**DOI:** 10.2471/BLT.22.289416

**Published:** 2023-05-01

**Authors:** Shatrunajay Shukla, Vivekanandan Kalaiselvan, Rajeev Singh Raghuvanshi

**Affiliations:** a Indian Pharmacopoeia Commission, Ministry of Health & Family Welfare, Government of India, Sector-23, Raj Nagar, Ghaziabad-201002, Uttar Pradesh, India.

## Abstract

Modern health-care facilities rely on medical devices and equipment. However, keeping up with the development of new technology is unfeasible for many health facilities, especially in low-resource settings. Thus, the demand for refurbished medical devices is increasing worldwide, especially in low- and middle-income countries. Refurbished medical devices are restored devices that are rebuilt to meet safety and performance requirements comparable to their condition when new, without changing the intended use of the original device. While new medical devices are controlled by well-established and stringent safety and quality regulations, a great variation in the regulations of refurbished medical devices exists across countries. Here we discuss the different regulations and practices specific to refurbished medical devices in countries of major markets. We also explore the opportunities and challenges for expanding the refurbished medical device market. Finally, we suggest that regulatory guidelines pertaining to the import, sale, labelling and use of a refurbished medical product are needed, and authorities should implement these guidelines to ensure a high quality and safety standard of refurbished devices.

## Résumé

Les établissements de soins de santé modernes dépendent d’équipements et dispositifs médicaux. Pour nombre de ces établissements, il est toutefois impossible de suivre l'évolution des nouvelles technologies, surtout dans les lieux manquant de ressources. La demande en dispositifs médicaux remis à neuf est donc en hausse partout dans le monde, en particulier dans les pays à revenu faible et intermédiaire. Il s'agit de dispositifs restaurés, remaniés pour répondre aux mêmes exigences de sécurité et de performances que lorsqu'ils sont neufs, sans que l'usage prévu du dispositif d'origine ne soit modifié. Alors que les dispositifs médicaux neufs sont soumis à des normes de qualité et de sécurité strictes et bien établies, leurs équivalents restaurés font l'objet de règles nettement plus variables d'un pays à l'autre. Dans le présent document, nous évoquons les différentes réglementations et pratiques spécifiques aux dispositifs médicaux remis à neuf dans les pays qui abritent les principaux marchés. Nous nous intéressons en outre aux opportunités et aux défis liés à un développement du marché des dispositifs médicaux remis à neuf. Enfin, nous suggérons l'adoption de lignes directrices réglementaires concernant l'importation, la vente, l'étiquetage et l'utilisation de tels dispositifs; ces lignes directrices sont à faire appliquer par les autorités afin de garantir les normes les plus élevées en matière de qualité et de sécurité.

## Resumen

Los centros sanitarios modernos dependen de dispositivos y equipos médicos. Sin embargo, mantenerse al día en el desarrollo de las nuevas tecnologías no es viable para muchos centros sanitarios, sobre todo en los de escasos recursos. Por este motivo, la demanda de dispositivos médicos renovados está aumentando en todo el mundo, especialmente en los países de ingresos bajos y medios. Los dispositivos médicos renovados son dispositivos restaurados que se reconstruyen para que cumplan unos requisitos de seguridad y rendimiento comparables a los que tenían cuando eran nuevos, sin cambiar el uso previsto del dispositivo original. Mientras que los dispositivos médicos nuevos están sujetos a reglamentos estrictos y bien establecidos en materia de seguridad y calidad, los reglamentos de los dispositivos médicos renovados varían mucho de un país a otro. En este artículo, se analizan los diferentes reglamentos y prácticas específicos de los dispositivos médicos renovados en los países de los principales mercados. También se exploran las oportunidades y los desafíos que plantea la expansión del mercado de dispositivos médicos renovados. Por último, se propone que se establezcan directrices reglamentarias relativas a la importación, venta, etiquetado y uso de los dispositivos médicos renovados y que las autoridades las apliquen para asegurar su calidad y seguridad.

## ملخص

تعتمد مرافق الرعاية الصحية الحديثة على الأجهزة والمعدات الطبية. وعلى الرغم من ذلك، فإن يتعذر مواكبة تطور التكنولوجيا الجديدة بالنسبة للعديد من المرافق الصحية، وخاصة في الأماكن منخفضة الموارد. وبالتالي، فإن الطلب على الأجهزة الطبية المجددة متزايد في جميع أنحاء العالم، وخاصة في الدول ذات الضغط المنخفض والدخل المتوسط. الأجهزة الطبية المجددة هي أجهزة مستردة تم إعادة بناؤها لتفي بمتطلبات السلامة والأداء المماثلة لحالتها عندما كانت جديدة، دون تغيير للاستخدام المزمع للجهاز الأصلي. بينما يتم التحكم في الأجهزة الطبية الجديدة من خلال لوائح راسخة وصارمة للسلامة والجودة، إلا أنه يوجد اختلاف كبير عبر الدول في لوائح الأجهزة الطبية المجددة. نحن نناقش هنا اللوائح والممارسات المختلفة الخاصة بالأجهزة الطبية المجددة في دول الأسواق الرئيسية. كما نستكشف أيضًا الفرص والتحديات أمام توسيع سوق الأجهزة الطبية المجددة. وأخيرًا، فإننا نشير إلى أن هناك حاجة إلى إرشادات تنظيمية تتعلق باستيراد، وبيع، وتمييز، واستخدام منتج طبي مجدد، وأنه يجب على السلطات تنفيذ هذه الإرشادات لضمان وجود مقياس للجودة العالية والسلامة للأجهزة المجددة.

## 摘要

现代卫生保健设施依赖于医疗器械和设备。然而，许多卫生设施无法跟上新技术的发展，尤其是在资源匮乏的环境中。因此，全球的翻新医疗器械需求越来越大，尤其是在中低收入国家。翻新医疗器械是指在不改变原器械预期用途的情况下，经修复可达到与其全新状态相当的安全和性能要求的器械。虽然新医疗器械相关安全和质量管制法规已经相当完善和严格，但各国的翻新医疗器械相关法规却存在很大差异。在此，我们讨论了主要市场国家/地区专门针对翻新医疗器械制定的不同法规和规范。我们还探讨了扩大翻新医疗器械市场的机遇和挑战。最后，我们建议制定与翻新医疗产品的进口、销售、贴标和使用相关的监管指南，并且当局应实施这些指南，以确保翻新器械能达到较高的质量和安全标准**。**

## Резюме

Современные медицинские учреждения полагаются на устройства медицинского назначения и оборудование. Однако многим медицинским учреждениям, особенно в условиях низких ресурсов, не под силу следить за развитием новых технологий. Таким образом, во всем мире, особенно в странах с низким и средним уровнем доходов, растет спрос на восстановленные устройства медицинского назначения. Восстановленные устройства медицинского назначения – это отремонтированные приборы, которые переделаны для соответствия требованиям безопасности и производительности, сопоставимым с их новым состоянием, без изменения предназначения исходного прибора. В то время как новые устройства медицинского назначения контролируются устоявшимися и строгими нормами безопасности и качества, в разных странах существуют большие различия в нормах регулирования восстановленных устройств медицинского назначения. В данном материале рассмотрены различные правила и практики, характерные для восстановленных устройств медицинского назначения в странах основных рынков. Также исследуются возможности и проблемы расширения рынка восстановленных устройств медицинского назначения. В заключение следует отметить, что необходимы нормативные рекомендации, касающиеся импорта, продажи, маркировки и использования восстановленных устройств медицинского назначения, а органы власти должны внедрить эти рекомендации для обеспечения высокого стандарта качества и безопасности восстановленных устройств.

## Introduction

Medical devices are an indispensable part of health-care systems and are used for prevention, diagnosis, treatment, monitoring, rehabilitation and palliation.^1^ Examples of such devices are: imaging equipment, in vitro diagnostic kits, implants, mobility aids, inhalers and medical equipment that support practitioners in performing clinical or surgical procedures.

Most managements of health-care facilities aspire to maintain a facility equipped with modern equipment and components that meet patients’ needs. However, medical device technology is evolving at such speed that newly released models are going out-of-date much faster.^2^ This technology development, as well as the cost of new devices, are making it unfeasible for managements to keep an up-to-date facility, especially in low-resource settings.

Quality equipment and components that work efficiently and are in good condition not only keep health-care professionals happy, but also minimize maintenance costs. If any medical device or component needs replacement, purchasing a new one is often the first solution that comes to mind. However, a new medical device or component can be expensive and hence unaffordable for facilities or service providers, especially in low- and middle-income countries. If health-care facilities lack the financial means to buy needed new equipment, they could purchase refurbished medical devices or refurbish the devices needing replacement. 

A refurbished medical device can be defined as a restored medical device rebuilt to meet safety and performance requirements that are comparable to its condition when new, without changing the intended use of the original device. Unlike used equipment, which is generally just cleaned up and sold as is, refurbished equipment involves replacement of worn-out parts, repair of mechanical and electrical components, reassembly, cosmetic touch, software updates, quality check, and testing and labelling updates. The Medical Device Authority at the Malaysian health ministry has provided a comprehensive definition of what a refurbished medical device is: “a medical device of which the whole or any part thereof has been rebuilt, whether or not using parts from one or more used medical devices of that same kind, so as to create a medical device that can be used for the purpose originally intended by the product owner of the original medical device, and which may have had the following work carried out on it: (i) stripping into component parts or sub-assemblies; (ii) checking their suitability for reuse; (iii) replacement of components or sub-assemblies not suitable for reuse; (iv) assembly of the reclaimed and/or replacement components or sub-assemblies; (v) testing of the assembled device against either original or revised release criteria; or (vi) identifying an assembled medical device as a refurbished medical device.”^3^

A typical refurbishment process includes dedicated stepwise activities. First, the client identifies and/or selects the used medical device based on the condition, service history, age of the device and availability of original spare parts and services. Second, authorized personnel disassemble, pack and ship the used device to the refurbishment facilities, usually to the original equipment manufacturer. The refurbishment of the device includes various processes, such as cleaning and disinfection, cosmetic refurbishment, mechanical and electrical refurbishment, system updates and configuration, quality testing and labelling. After the manufacturer has returned the refurbished device to the client’s location, an authorized agent from the refurbishment provider re-installs the device and performs a standard system performance check, similar to a check of a new system. The agent also provides application training for device operators. The manufacturer usually provides warranty and after-sales services of the refurbished devices.

## Refurbished medical device market

The global refurbished medical device market is projected to increase from 12.1 billion United States dollars (US$) in 2020 to US$ 21.2 billion in 2025, with an annual average growth rate of 11.8%.^4^ Increasing privatization of the health-care sector to meet demands for health care,^5^^,^^6^ especially in low- and middle-income countries, along with pressing requirements of low-cost medical services, will foster the refurbished medical device market. In addition, the global rise in the prevalence of acute and chronic illness will increase the demand for health care, and further propel the growth in the demand for refurbished medical devices.^7^^,^^8^

Factors driving the growth of refurbished or reprocessed medical equipment markets are: (i) an increasing demand for cost–effective medical devices by small-scale private hospitals and clinics; (ii) health-care providers’ large inventories of used and/or old medical devices; (iii) internet platforms easing the purchase of refurbished medical equipment; (iv) growing preference for eco-friendly products; (v) an increasing number of diagnostic centres and hospitals; and (vi) the growing opportunities to provide refurbished devices in emerging economies.^9^^–^^11^ To meet the demand, key manufacturers such as GE HealthCare, Siemens Healthineers and Koninklijke Philips N.V. are increasing their refurbishing services of their equipment. While demand is growing, factors such as stringent regulations on the import and use of refurbished medical devices; lack of standardized policies for the use and sale of these devices; increase in low-cost new medical devices; and negative perceptions about the quality of refurbished medical devices are expected to restrain the market growth.

### Market size

According to reports, North America holds the largest market share of the refurbished medical equipment market, followed by Europe and the Asia Pacific region. An estimated 70% of all refurbished imaging medical devices are sold in United States of America (46%) and the European Union (EU; 24%).^4^^,^^12^ However, the Asia Pacific market is expected to have the highest annual growth rate in the coming years.^4^^,^^13^ This growth can be attributed to the large population in the region, increasing privatization of the health-care sector, and a high demand for refurbished medical equipment by hospitals and clinics with constrained resources. 

## National regulations

National variations exist in the regulations of refurbishment of medical devices. Major markets, such as the EU and the USA, accept sale and import of refurbished medical devices, while other countries do not. To demonstrate the variety of regulations across countries, we here provide examples of regulations in countries of major markets as well as other countries.

### EU

The medical device directives and the medical device regulations do not define refurbishment. Up until 26 May 2021, refurbished medical devices were regulated similarly to new medical devices by the medical device directives 93/42/EEC^14^, 90/385/EEC^15^ and 98/79/EC.^16^ These directives have been replaced by the medical device regulation (EU) 2017/745,^17^ which is applicable for new as well as refurbished medical devices. The definition “fully refurbishing” used in the medical device regulation is similar to the term “remanufacturing” used in the United States regulation.^18^ Fully refurbishing refers to the complete rebuilding of a device already placed on the market or put into service, or the making of a new device from used devices, to bring the refurbished device into conformity with (EU) 2017/745, combined with the assignment of a new lifetime to the refurbished device. No country-specific regulations exist among EU countries.

### India

The import of used critical care medical equipment for reuse in India is regulated through Hazardous and other Wastes (Management, Handling and Transboundary Movement) Rules, 2016.^19^ However, no clear definition or specific guidelines for refurbishment of medical devices are available in India. Critical care medical equipment means life-saving equipment, and includes equipment specified by the Ministry of Health and Family Welfare. Box 1 lists the 27 critical care medical devices that are banned from import and reuse under Schedule VI (that is, hazardous and other wastes prohibited for import) of Hazardous and other Wastes (Management, Handling and Transboundary Movement) Rules, 2016.

Box 1 **Equipment that are banned under Schedule VI of Hazardous and other Wastes (Management, Handling and Transboundary Movement) Rules, 2016, India^19^**

Bedside monitor for intensive care units; monitor for high dependency unit; ventilator; non-invasive ventilator; fibreoptic bronchoscope; head-end panel; defibrillator; arterial blood gas machine; crash and/or resuscitation trolley; pulse oximeter (small units); medical grade refrigerator; haemodialysis machine; continuous renal replacement therapy; cardiac output, systemic vascular resistance and central venous oxygen saturation monitor; intermittent leg compressing machine; intubating videoscope; intensive care unit dedicated ultrasound and echo machine; bedside X-ray machine; rigid cervical spine collar; different sizes of Ambu® masks; intra-aortic balloon pump; infusion pump; syringe pump; shock-proof bed for intensive care unit; over-bed table; and glucometer.

### Japan

Japanese authorities allow the import and sale of refurbished medical devices, including spare parts. Refurbished medical devices are regarded as a subset of used medical devices. No specific definition or guidelines regarding refurbishment are available. However, refurbishment should be performed in the facilities which meet Japanese good manufacturing practices, and the refurbishment should meet the quality management system requirements stipulated in the Ministerial Ordinance on Standards for Manufacturing Control and Quality Control for Medical Devices and In Vitro Diagnostics (MO 169),^20^ and the refurbished medical device should be shipped from the original equipment manufacturer. The requirements applicable for new medical devices, that is, JIS T 0601–1:2017,^21^ are also applicable for used medical devices or repair of medical devices.

### United Kingdom

In the United Kingdom of Great Britain and Northern Ireland, as of now, regulations are similar to those applicable in EU countries. CE-marked devices (that is, products that meet European health, safety, and environmental protection requirements) are acceptable in the United Kingdom market until 30 June 2023. From 1 July 2023, devices that are placed on the British market will need to conform with the United Kingdom conformity-assessed marking requirements.^22^

### USA

The United States Food and Drug Administration (FDA) does not define the term refurbished medical device, but considers a refurbishment process as a servicing activity. However, there is lack of clarity between servicing and remanufacturing of a medical device. According to an FDA document,^18^ servicing is the repair and/or preventive or routine maintenance of one or more parts in a finished device, after distribution, for purposes of returning it to the safety and performance specifications established by the original equipment manufacturer, and to meet its original intended use. Servicing cannot change the intended use(s) of the device from its original purpose(s). While remanufacturing is the processing, conditioning, renovating, repackaging, restoring, or any other act done to a finished device that significantly changes the finished device's performance or safety specifications, or intended use.^18^ Refurbishment includes reconditioning, repair, installation of certain software and/or hardware updates that do not change the intended use of the original device, and replacement of worn parts. Refurbished medical devices are permitted to be sold in and imported to the USA market. A refurbished medical device, similar to a new medical device, must possess a relevant premarket clearance or authorization to be placed on the USA market, e.g. 510(k) premarket notification for class II (that is, devices with moderate to high safety risk) or a premarket approval for class III (that is, devices with high safety risk).^23^ Import of refurbished devices to the USA market is subject to FDA registration of the source site (that is, where refurbishment is performed) as foreign exporter.

### Other national regulations

In some countries, such as Kenya, the Philippines, Republic of Korea, Sudan and Singapore, we were unable to find specific guidelines for refurbishment of medical devices. However, import of refurbished or reprocessed medical devices is subject to fulfilment of registration requirements and quality testing.^24^

Malaysian authorities use their *Good refurbishment practice of medical devices* to regulate sales and import of refurbished medical devices.^25^ In 2022, the Pakistani drug regulatory authority amended their Medical Devices Rules 2017 to define the terms refurbished medical device and refurbishment. Regulations pertaining to refurbished medical devices shall be regulated as per procedures and standards prescribed in amendments in Medical Device Rules 2017.^26^

In Indonesia, Thailand and Viet Nam, import of refurbished medical devices and spare parts is banned or not permitted. In some Eastern Mediterranean countries such as Egypt and Morocco, import and sale of used or second-hand and refurbished medical devices is also banned. Importers must present an original certificate from the manufacturer indicating the manufacturing year of the medical device, and that it is new and safe to use.^24^^,^^27^

In some Latin American countries such as Venezuela, Peru and Ecuador, import of refurbished medical devices is only allowed in the private sector. However, in Argentina, Brazil and Uruguay, imports of refurbished medical devices are allowed, but subject to a technical assessment certificate or technical documentation, respective ministerial approval and selective conditions.^28^

## Donations

Health-care systems in low- and middle-income countries often have limited access to common medical devices and equipment. New medical devices are hard to procure in these countries due to economic and political constraints. Thus, health-care systems rely considerably on donations from international donors or foreign governments. In some of these countries, nearly 80% of the health-care equipment supply is received through donations or funding from international donors or high-income countries.^29^ While most of the donations are given with the good intent to strengthen health-care systems and improve the well-being of the people being served, only 10–30% of donated medical equipment become operational in low- and middle-income countries.^30^^,^^31^ Reasons for unused medical equipment include mismanagement in the technology acquisition process, and lack of user training and effective technical support.^29^ Considering this scenario, the World Health Organization (WHO) has published the document *Medical device donations: considerations for solicitation and provision*, which describes issues and challenges surrounding medical device donations.^29^ This document provides considerations and best practices that may be useful for improving the quality of medical equipment donations, and provide maximum benefit to all stakeholders.

## Challenges

The refurbished medical device sector in low- and middle-income countries is facing several challenges. First, the dominance of major multinational companies, such as GE HealthCare, Siemens Healthineers, Koninklijke Philips N.V. and EVERX. These companies are already present in major markets of low- and middle-income countries and they have an established supply-chain network. Hence, there is not much room for domestic companies. Second, the sector is unorganized, allowing companies that do substandard refurbishing to charge less for reconditioned equipment than their organized counterparts. Third, many people perceive used or pre-owned products to be of inferior quality and hence may refrain from buying refurbished products. Finally, most consumers import refurbished medical equipment without after-sales service. This shortcoming adds to the cost of refurbished medical devices by roughly half of the total cost of such devices.^32^

## Opportunities

Several opportunities exist for the refurbished medical device sector. First, the market for refurbished medical equipment is growing in low- and middle-income countries. Companies can seize this opportunity and increase their share in this market. Second, no major domestic players are present in the refurbishment market in low- and middle-income countries. An already-established domestic medical equipment company can profit by diversifying into this sector. Third, the small town and rural markets are still untapped. Although affordability is a major issue, collaborations with state and central governments can be used to develop this market. Finally, companies can establish national centres providing customers with after-sales services, and thereby increase the possibility of gaining the trust of customers in refurbished products.

## Conclusion

Although refurbished medical devices save materials and resources, regulating the refurbished devices market is important to ensure the quality and safety of these products. Therefore, a regulatory framework or appropriate standards for refurbishing medical devices and guidelines pertaining to their import, sale, labelling and use are needed. Standards such as IEC 63077:2019,^33^ which describes and defines the process of refurbishment of used medical imaging equipment, and ISO 13485:2016,^34^ which specifies requirements for a quality management system for medical devices and related services, could be good starting points for the development of standards and guidelines. WHO is also finalizing the guidelines for refurbishing processes, labelling requirements and regulations in the revised version of *WHO Global Model Regulatory Framework for Medical Devices including in vitro diagnostic medical devices*.^35^ If such guidelines are developed and implemented, possible harm to patients or users due to refurbished device malfunction can be reduced.
